# 324. Epidemiological Trends of Streptococcus pneumoniae Pneumonia in Argentine Children: Serotype Distribution, Antibiotic Resistance, and Vaccine Coverage Gaps (2022-2024)

**DOI:** 10.1093/ofid/ofaf695.113

**Published:** 2026-01-11

**Authors:** Jonathan C Zintgraff, Paula Gagetti, Nahuel Sanchez Eluchans, Paulina Marchetti, Maria Alicia Moscoloni, Claudia Lara, Alejandra Corso

**Affiliations:** INEI-ANLIS DR CARLOS G MALBRAN, CABA, Ciudad Autonoma de Buenos Aires, Argentina; INEI-ANLIS DR CARLOS G MALBRAN, CABA, Ciudad Autonoma de Buenos Aires, Argentina; INEI-ANLIS DR CARLOS G MALBRAN, CABA, Ciudad Autonoma de Buenos Aires, Argentina; INEI-ANLIS DR CARLOS G MALBRAN, CABA, Ciudad Autonoma de Buenos Aires, Argentina; INEI-ANLIS DR CARLOS G MALBRAN, CABA, Ciudad Autonoma de Buenos Aires, Argentina; Instituto Nacional de Enfermedades Infecciosas ANLIS "Dr Carlos G Malbran", Ciudad Autónoma de Buenos Aires, Ciudad Autonoma de Buenos Aires, Argentina; Instituto de Enfermedades Infecciosas ANLIS "Dr Carlos G Mabrán", Buenos Aires, Buenos Aires, Argentina

## Abstract

**Background:**

Invasive pneumococcal pneumonia (IPP) remains a leading cause of morbidity and mortality in children. Argentina incorporated PCV13 into its National Immunization Program in 2012, significantly reducing IPP burden. However, ongoing epidemiological surveillance remains critical to monitor circulating serotypes and their evolving resistance pattern. Objectives: To analyze the serotype distribution and antimicrobial resistance profile in cases of IPP in children under 11 years of age in Argentina, during 2022-2024.

**Figure 1:**
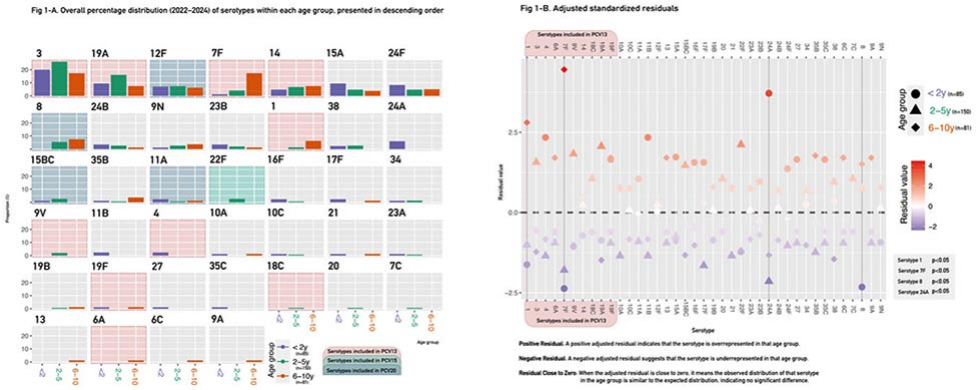
Figure 1-A presents the overall percentage distribution (2022–2024) of serotypes within each age group, arranged in descending order according to their prevalence in this study. Figure 1-B displays the adjusted standardized residuals, highlighting serotypes with statistically significant p-values.

**Figure 2:**
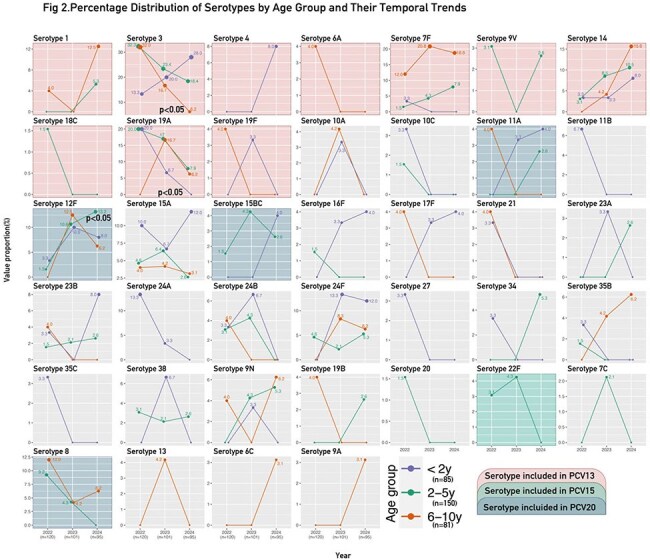
The analysis of IPP cases by serotype, age group, and year reveals notable temporal trends that may reflect changes in pneumococcal epidemiology.

**Methods:**

The study included 316 isolates obtained from patients under 11 years old, collected from 78 hospitals across 23 jurisdictions. Serotyping was performed by Quellung and MICs by agar dilution. Isolates were stratified into three age groups (< 2 years [n=85], 2-5 years [n=150], and 6-10 years [n=81]) and by clinical presentation (pneumonia with effusion [n=135] vs without effusion [n=179]). MIC analysis was conducted for the < 2 years (n=79) and 2-5 years (n=147) groups.

**Figure 3:**
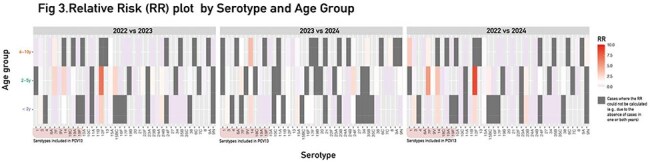
Analysis of Relative Risk (RR) by Serotype and Age Group. To better understand these trends, a heatmap was created to visualize the relative risk (RR) for each serotype across age groups and years (Fig3). The heatmap highlights serotypes with significant increases or decreases in risk, making it easier to identify patterns over time.

**Figure 4:**
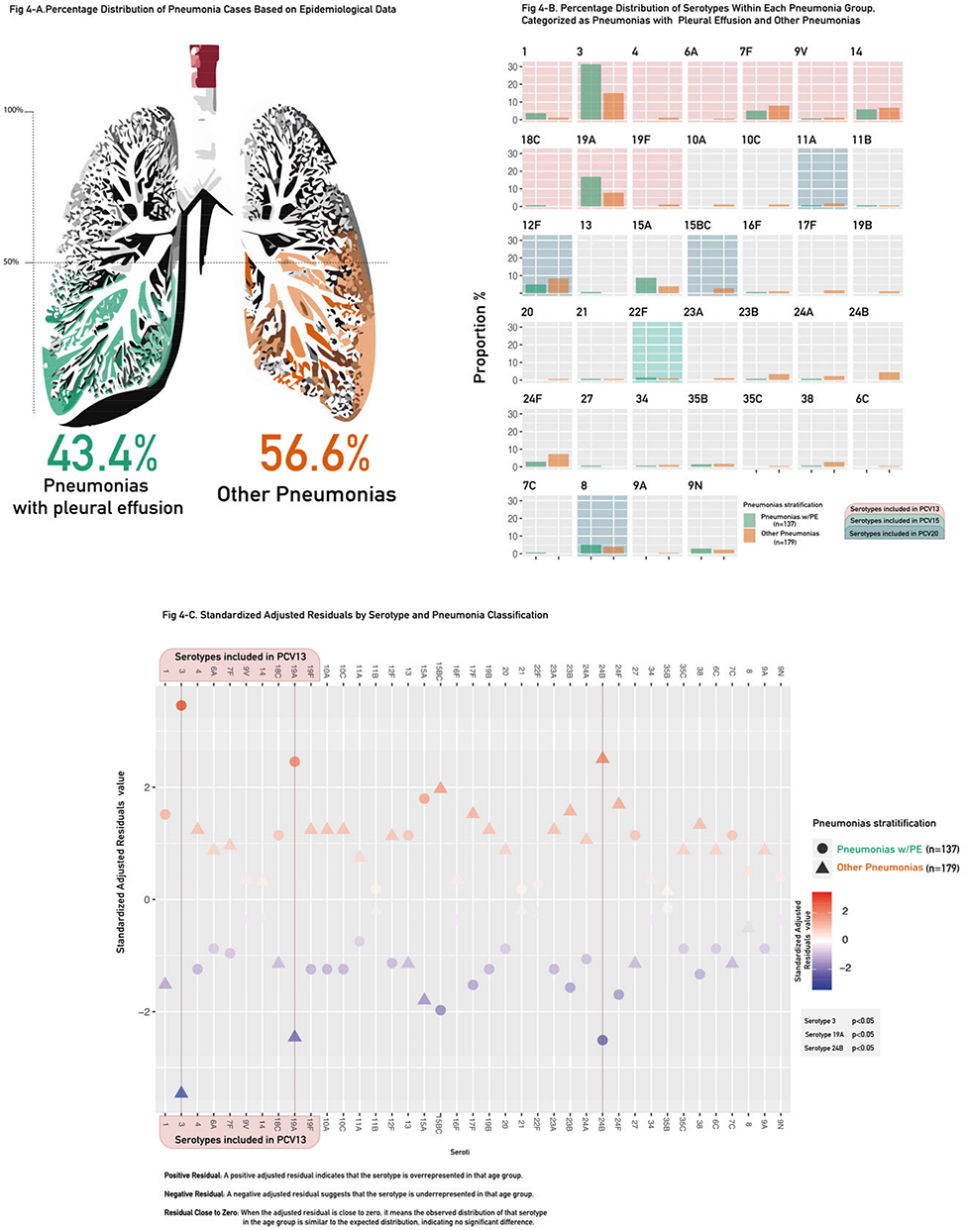
Figure 4-A presents a graphical representation of case distribution percentages, derived from epidemiological data collected through standardized case report forms accompanying each bacterial isolate. Panel B highlights the distribution of serotypes within each pneumonia category. Notably, only three serotypes demonstrated significant associations (p < 0.05): serotypes 3 and 19A were linked to pleural effusion, while serotype 24B was associated with other pneumonias. Finally, Panel C provides the SAR for further analysis.

**Results:**

Serotypes 3, 19A, 14, 12F, 15A, and 24F predominated overall, with age-specific variations: 7F and1 in 6-10-years, 24A exclusively in < 2 years, and serotype 8 in 2-10-years. Serotypes 3,19A were strongly associated with pleural effusion (p< 0.05), while 24B linked to non-effusion pneumonia. Non-susceptibility (I+R) to: penicillin non-meningitis 6.2%, cefotaxime non-meningitis 2.7%, amoxicillin 5.8%, erythromycin 30.1%, clindamycin 24.8%, tetracycline/doxycycline 45.6% and levofloxacin 0%. MDR strains (24.3%) were primarily serotypes 19A and 24F/A/B (68%, p< 0.01).

**Conclusion:**

No significant differences in antimicrobial resistance patterns were observed between ages. The association of serotypes 3 and 19A with pleural effusion may reflect their enhanced virulence profiles. The increased prevalence of serotypes 7F and 1 in older children (6-10 years) likely indicates waning immunity from PCV13 vaccination over time. Penicillin and amoxicillin maintain their status as first-line therapeutic options for pneumococcal pneumonia, supported by their sustained low non-susceptibility rates.

**Disclosures:**

All Authors: No reported disclosures

